# Comparison of CT-Number and Gray Scale Value of Different Dental Materials and Hard Tissues in CT and CBCT

**Published:** 2014-10-07

**Authors:** Naghmeh Emadi, Yaser Safi, Alireza Akbarzadeh Bagheban, Saeed Asgary

**Affiliations:** a *Dental Research Center, Research Institute of Dental Sciences, Shahid Beheshti University of Medical Sciences, Tehran, Iran; *; b *Department of Oral and Maxillofacial Radiology, Dental School, Shahid Beheshti University of Medical Sciences, Tehran, Iran;*; c *Iranian Center for Endodontic Research, Research Institute of Dental Sciences, Shahid Beheshti University of Medical Sciences, Tehran, Iran;*; d *Department of Basic Sciences, School of Rehabilitation Sciences, Shahid Beheshti University of Medical Sciences, Tehran, Iran*

**Keywords:** Computed Tomography, Cone-Beam Computed Tomography, Dental Materials, Dentistry, Gray Scale Value, Hounsfield Unit, Spiral Computed Tomography

## Abstract

**Introduction:** Computed tomography (CT) and cone-beam CT (CBCT) are valuable diagnostic aids for many clinical applications. This study was designed to compare the gray scale value (GSV) and Hounsfield unit (HU) of selected dental materials and various hard tissues using CT or CBCT. **Methods and Materials:** Three samples of all test materials including amalgam (AM), composite resin (CR), glass ionomer (GI), zinc-oxide eugenol (ZOE), calcium-enriched mixture (CEM) cement, AH-26 root canal sealer (AH-26), gutta-percha (GP), Coltosol (Col), Dycal (DL), mineral trioxide aggregate (MTA), zinc phosphate (ZP), and polycarbonate cement (PC) were prepared and scanned together with samples of bone, dentin and enamel using two CBCT devices, Scanora 3D (S3D) and NewTom VGi (NTV) and a spiral CT (SCT) scanner (Somatom Emotion 16 multislice spiral CT);. Subsequently, the HU and GSV values were determined and evaluated. The data were analyzed by the Kruskal-Wallis and Mann-Whitney U tests. The level of significance was determined at 0.05. **Results: **There were significant differences among the three different scanners (*P*<0.05). The differences between HU/GSV values of 12 selected dental materials using NTV was significant (*P*<0.05) and for S3D and SCT was insignificant (*P*>0.05). All tested materials showed maximum values in S3D and SCT (3094 and 3071, respectively); however, bone and dentin showed low/medium values (*P*<0.05). In contrast, the tested materials and tissues showed a range of values in NTV (366 to15383; *P*<0.05). **Conclusion:** Scanner system can influence the obtained HU/GSV of dental materials. NTV can discriminate various dental materials, in contrast to S3D/SCT scanners. NTV may be a more useful diagnostic aid for clinical practice.

## Introduction

American Dental Association (ADA) Council on Dental Materials and Devices released a statement in 1981 that declared the optical density as a desirable requirement for restorative materials [1]. It is generally accepted that dental materials should have sufficient radiopacity to be detectable against enamel and dentin backgrounds in dental imaging. This characteristic is also helpful for the assessment of restorations as well as endodontic fillings, detection of secondary caries, evaluation of marginal defects, restoration contour and other defects on radiographs [2]. According to ISO standard the commonly used techniques for the assessment of radiographic density include digital radiography with occlusal film using aluminum step-wedge [3, 4].

Computed tomography (CT) was introduced in 1970 by Hounsfield [5]. For image display each pixel is assigned a CT number (Hounsfield units; HU) representing tissue density. HU ranges from -1000 to +1000; however, some newer CT scans have a range of up to 4000 HU [6]. The technology was introduced in 1978 for bone density [7]. Several studies have evaluated this object and their method/results are considered to be the gold standard for evaluation of the density of tissues [8].

**Table 1:** Test materials and their composition

**Products**	**Components**
**Amalgam **	Ag=49%, Sn=29%, Cu=22%, Hg=29%
**Composite Resin **	Bis-GMA, TEGDMA, zirconia, silica fillers
**Glass Ionomer **	Aluminum fluorosilicate glass, ZnO (powder), polyacrylic acid, HEMA, water, photo initiator (liquid)
**Zinc-Oxide Eugenol **	Zinc-oxide (powder), eugenol or carboxylic acid and inert filler (liquid)
**CEM Cement**	Calcium-silicate, calcium-phosphate, calcium-oxide, calcium-salts, barium-sulfate and zirconium
**AH-26 Sealer **	Bismuth-oxide, calcium-hydroxide, hexamethylenetetramine, titanium-dioxide and bis-phenol
**Gutta-Percha**	Poly-isoprene rubber, zinc-oxide, barium-sulfate, coloring agent
**Coltosol**	Zinc-oxide, zinc-sulphate, calcium-sulphate, dibutyl-phethalate, polyvinyl-acetate chloride copolymer, peppermint
**Dycal**	Butyene glycol disaliclate, zinc-oxide, calcium-phosphate, calcium-tungstate, iron-oxide (base), calcium-hydroxide, sulfonamide, zinc-oxide, titanium-dioxide, zinc-stearate, iron-oxide pigments (catalyst)
**MTA**	Portland cement, bismuth-oxide and gypsum
**Zinc Phosphate Cement **	Zinc-oxide, magnesium-oxide (powder), o-phosphoric acid (liquid)
**Poly Carbonate Cement **	Zn, Mg and Al oxides, boric acid (powder), acrylic acid, maleic acid anhydride, distilled water (liquid)

**Table 2:** Scanner settings and software applied for spiral computed tomography (SCT) and two cone-beam computed tomography (CBCT) devices

**Equipment setting**	**Somatom emotion 16 **	**Scanora 3D **	**NewTom VGi**
**Kilo voltage peak**	130 kVp	90 kVp	110 kVp
**Milliamperage **	139 mA	12 mA	0.56 mA
**Field of view**	50×50 mm	75×100 mm	80×120 mm
**Software**	Syngo FastView	OnDemand 3D	NTT viewer

Cone-beam computed tomography (CBCT) was first used for angiography in 1982 and since the late 1990s it has also been utilized for dentistry [6]. There are some controversies surrounding the reliability of density measurement by CBCT systems; however, the studies have mostly focused on bone density and imaging parameters [9, 10]. To date, no study has evaluated the density of endodontic and restorative materials using CBCT.

The objective of the present laboratory study was to compare the CT number (HU) and gray scale value (GSV) of selected dental materials as well as various hard tissues using two CBCT devices and one CT scanner.

## Methods and Materials

***Preparation of samples***

Holes measuring 3 mm in diameter and 2 mm in height were prepared in 36 resin molds (3 molds for each test material). All the dental materials including amalgam (AM; Cinalux, Faghihi Dental Co., Iran), Composite Resin (CR; Valux plus, 3M Dental Products, USA), Glass Ionomer (GI; Fuji II, GC Corporation, Tokyo, Japan), Zinc-Oxide Eugenol (ZOE; Kemdent; Associated Dental Products, UK), Calcium-Enriched Mixture (CEM Cement; BioniqueDent, Tehran, Iran), AH-26 sealer (AH-26; Dentsply, Tulsa Dental, Tulsa, OK, USA), gutta-percha (GP; Ariadent, Tehran, Iran), Coltosol (Col; Ariadent, Tehran, Iran), Dycal (DL; Dentsply, Tulsa Dental, Tulsa, OK), ProRoot MTA (MTA; Dentsply, Tulsa Dental, Tulsa, OK, USA), zinc-phosphate cement (ZP; Hoffmann Dental Manufaktur GmbH, Berlin, Germany) and polycarbonate cement (PC; Harvard Dental GmbH, Berlin, Germany) were prepared according to the manufactures’ instructions and placed in molds. Three extracted premolars, as well as cortical/spongy bone (from the posterior of maxilla) were scanned to evaluate the density of enamel, dentin and bone. The components of the materials are listed in Table 1. 

***Scanning procedure***

Scans were obtained from tested materials using the NewTom VGi (NTV; QR SRL Co., Verona, Italy), Scanora 3D (S3D; Soredex, Helsinki, Finland) and multislice spiral CT scanner (SCT; Somatom Emotion; Siemens Medical Solutions, Forchheim, Germany) (Table 2). Density of various dental materials was determined using the GSV and HU obtained from CBCT and SCT scanners, respectively.

***Image analysis***

To overcome the probable inhomogeneous nature of the samples, each final HU/GSV of tested material was based on the average of 36 records; each sample (*n*=3) was divided into four parts, and three measurements of HU/GSV were recorded for each part. The data were collected and recorded by one radiologist. The softwares used for image analysis included Syngo FastView (AG 2004; Siemens, Munich, Germany), OnDemand 3D (Cybermed Inc, Irvine, CA) and NTT viewer (NTT Software Corporation, Yokohama, Japan) for SCT, S3D and NTV, respectively.

***Statistical analysis***

The collected data were analyzed statistically with the Kruskal-Wallis test and Mann-Whitney U test (SPSS version 16.0, SPSS, Chicago, IL, USA). The level of significance was set at 0.05.

**Table 3:** Mean, standard error (SE), Minimum (Min) and Maximum (Max) of gray scale values (GSV) of experimental materials

**Materials**	**Mean**	**SE**	**Min**	**Max**
**Amalgam**	15383.0	0.0	15383.0	15383.0
**AH-26 Sealer**	15383.0	0.0	15383.0	15383.0
**Zinc Phosphate Cement **	13987.3	42.8	12789.0	14322.0
**Gutta-Percha **	13964.4	47.2	13452.0	14424.0
**Zinc-Oxide Eugenol **	13860.8	67.8	13156.0	14632.0
**Poly Carbonate Cement **	12661.9	44.4	12024.0	13045.0
**Mineral Trioxide Aggregate **	11270.3	36.5	10981.0	11956.0
**Coltosol **	8616.8	31.4	8142.0	8971.0
**Calcium-Enriched Mixture **	8506.5	22.9	8307.0	8876.0
**Composite Resin**	6896.0	14.5	6719.0	7076.0
**Glass Ionomer **	6391.3	14.7	6207.0	6543.0
**Dycal**	4635.0	18.2	4423.0	4906.0
**Enamel**	4358.9	11.3	4187.0	4490.0
**Dentin**	2583.7	17.2	2403.0	2764.0
**Cortical bone**	1766.2	34.6	1470.0	2134.0
**Spongy bone**	541.6	17.8	366.0	745.0

**Table 4:** *P*-value obtained by the Mann-Whitney U test for pairwise comparison of groups

**Materials**	**GI**	**ZOE**	**CEM**	**GP**	**CR**	**DL**	**MTA**	**ZP**	**PC**
**Composite Resin (CR) **	*	*	*	*	*	*	*	*	*
**Glass Ionomer (GI) **	-	*	*	*	*	*	*	*	*
**Zinc-Oxide Eugenol (ZOE) **	*	-	*	¥	*	*	*	¥	*
**Calcium-Enriched Mixture (CEM) **	*	*	-	*	*	*	*	*	*
**Gutta-Percha (GP) **	*	*	*	-	*	*	*	¥	*
**Coltosol (Col) **	*	*	*	*	-	*	*	*	*
**Dycal (DL)**	*	*	*	*	*	-	*	*	*
**Mineral Trioxide Aggregate (MTA)**	*	*	*	*	*	*	-	*	*
**Zinc Phosphate Cement (ZP) **	*	*	*	*	*	*	*	-	*
**Poly Carbonate Cement (PC)**	*	*	*	*	*	*	*	*	-

(*) indicates significant differences (P<0.001); and (¥) indicates nonsignificant differences (P>0.05)

## Results

AM and AH-26 had the highest GSV among the tested dental materials. ZP, GP and ZOE stood in the second rank, followed by PC and MTA. Except for cortical bone, spongy bone and dentin, HU/GSV of tested materials in SCT scan and S3D was equal to the maximum value demonstrated by the devices (3071 and 3094, respectively). In NTV, AM and AH-26 had the highest GSV, which was equivalent to 15383. The obtained values of other tested materials were all below this limit. In samples with HU/GSV below the maximum threshold of the device, S3D and NTV revealed no significant differences in comparison with SCT (*P*>0.05).

The result of the tested groups showed inequality of variances, which was largely due to the absence of disparity in AM and AH-26 groups, the nonparametric Kruskal-Wallis test were used for the inter-group comparisons. Significant differences were observed between the groups scanned with the NTV device in GSV (*P*<0.05). Table 3 demonstrates the GSV of the 16 experimental materials.

Pairwise comparison of groups with nonparametric Mann-Whitney U test detected statistically significant differences between majorities of tested materials (Table 4). Due to the absence of variation in the AM and AH-26 groups, these two materials were not compared with others.

## Discussion

In the present study, we used one CT device (SCT) and two CBCT systems (NTV and S3D) for determination of HU/GSV of various dental materials. Our results clearly demonstrated that the choice of imaging device could affect the measured HU/GSV of a dental material. Currently, conventional intraoral radiography is the most commonly used system for the evaluation of the technical quality of the orthograde as well as retrograde filling materials; however, CBCT technology has recently gained popularity in endodontic profession [11]. Contrary to S3D and SCT scanners, the NTV device could precisely discriminate various dental materials.

According to ANSI/ISO standards, radiopacity of endodontic materials using conventional radiography should be higher than tooth structure and comparable with that of the determined thickness of aluminum. For example, the radiopacity of 1 mm of dentin and aluminum is equal but an endodontic sealer should present radiopacity correspond to at least 3 mm aluminum; however, differences in the step-wedge of aluminum alloy as well as device/test setting (*i.e*. exposure time, kVp, mA, focal film distance, film speed, imaging technique, and developing process) can affect the outcome and lead to inhomogeneous results [12]. On the other hand, there are no specific standards for HU/GSV of dental materials using CT/CBCT up to the present moment.

Several studies have evaluated the GSV and bone density measurements by CBCT and have yielded conflicting results [8, 13, 14]. Some reported a higher bone density value obtained by CBCT than by SCT and found it to be unreliable [13]. However, other investigations achieved a high level of correlation (*r=*0.965) of density between CBCT and SCT [15]. Furthermore, evidence suggested that HU value of the respective tissues could be obtained from the GSV by using linear attenuation coefficient and its application in an equation and reported small differences with actual HU values [16].

Our results demonstrated that using NTV, AM and AH-26 root canal sealer had the highest GSV among the tested dental materials. ZP cement, GP and ZOE took the second rank, followed by PC cement and MTA. Similar results have been reported from some recent studies by the step-wedge method [17].

## Conclusion

Under the conditions of this study, the results demonstrated that the tested dental materials had different GSVs with the NewTom VGi CBCT device; compared to Scanora 3D CBCT and Somatom Emotion spiral CT scanner. It appears that NewTom VGi is a useful diagnostic imaging system for detecting the various dental materials.
